# Enhancing the performance of preclinical international medical students’ in the subjects “medical psychology and medical sociology” through a peer-led revision course

**DOI:** 10.3205/zma001853

**Published:** 2026-06-15

**Authors:** Julia Sgrott, Christoph Nikendei, Hans-Christoph Friederich, Ivo Dönnhoff

**Affiliations:** 1 University of Heidelberg, Department of General Internal Medicine, Psychosomatics and Psychotherapy, Heidelberg, Germany; 2 Heidelberg’s Tutorial for International Medical Students (HeiTiMed), Heidelberg, Germany; 3 University Hospital of Heidelberg, DZPG – German Center for Mental Health Mannheim-Heidelberg-Ulm, Heidelberg, Germany

**Keywords:** peer-assisted learning, PAL, international students, revision course, social congruence, cognitive congruence

## Abstract

**Objective:**

When moving to a new country for their university studies, international students are confronted with numerous difficulties in their social and academic life. Various projects have been developed to support international students in facing these difficulties, such as peer-assisted learning tutorials. In this study, our goal was to evaluate preclinical international medical students’ objective improvement of knowledge after attending a three-day peer-led revision course for the subjects ‘medical psychology and medical sociology’.

**Methods:**

The sample consisted of n=30 international students. The objective improvement of knowledge was quantified by the amount of correctly answered questions in the post-test compared to the pre-test. Students were randomly separated into groups based on which test version (A or B) was written in the pre-test. For the comparison between pre- and post-times and between groups, a two-way ANOVA was calculated.

**Results:**

International students significantly improved their knowledge, as reflected in higher post-test scores compared to pre-test scores (F_1,22_=13.470, p=0.001).

**Conclusion:**

International students showed an improvement in their knowledge, suggesting a potential benefit from participating in a peer-led revision course. Peer-led tutorial programs for international students should be implemented further in medical faculties as means of supporting these students academically and socially.

## Zusammenfassung

**Ziel:**

Wenn internationale Studierende für ihr Studium in ein neues Land ziehen, werden sie mit zahlreichen Schwierigkeiten in ihrem sozialen und akademischen Leben konfrontiert. Zur Unterstützung internationaler Studierender bei der Bewältigung dieser Schwierigkeiten wurden verschiedene Projekte entwickelt, wie beispielsweise Peer-Assisted Learning (PAL) basierte Tutorien. Das Ziel dieser Studie war es, die objektive Wissensverbesserung vorklinischer internationaler Medizinstudierender nach der Teilnahme an einem dreitägigen, Peer-geleiteten Repetitorium in den Fächern „Medizinische Psychologie und Medizinische Soziologie“ zu evaluieren.

**Methoden:**

Die Stichprobe bestand aus n = 30 internationalen Studierenden. Die objektive Wissensverbesserung wurde anhand der Differenz an korrekt beantworteten Fragen im Prä- und Post-Test quantifiziert. Die Studierenden wurden randomisiert in Gruppen eingeteilt, je nachdem, welche Testversion (A oder B) sie im Prä-Test absolviert hatten. Für den Vergleich zwischen Prä- und Post-Zeitpunkt sowie zwischen beiden Gruppen wurde eine zweiseitige ANOVA berechnet.

**Ergebnisse:**

Die internationalen Studierenden verbesserten ihr Wissen signifikant, was sich in besseren Post-Test-Ergebnissen im Vergleich zu den Prä-Test-Ergebnissen widerspiegelte (F_1,22_=13,470, p=0,001).

**Schlussfolgerung:**

Internationale Studierende zeigten eine Verbesserung ihres Wissenstands, was auf eine positive Wirkung der Teilnahme an einem Peer-geleiteten Repetitorium hindeutet. Peer-geleitete Repetitorien für internationale Studierende sollten an medizinischen Fakultäten weiter ausgebaut werden, um internationale Studierende akademisch und sozial zu unterstützen.

## 1. Introduction

In Germany, “international students” are defined as students who accomplish their university entry qualification in another country and come to Germany for university education [1]. With around 292 000 international students attending German universities in 2023, Germany continues to be a very attractive country for those who seek to complete their higher education abroad. Around 5% of these international students are enrolled in medicine, representing around 13% of the total of medical students in Germany [2]. Several authors [3], [4], [5] have previously described the hurdles that international medical students face and must overcome when pursuing their university degree. These hurdles range from lack of social and financial support [3], [5] to personal distress [4], [6], and language and cultural barriers [7], [8]. These challenges may also contribute to the observed lower academic performance of international medical students in written, oral, and practical examinations [9]. Huhn et al. point out that international students in German-speaking regions achieved lower results in the oral state examination when compared to German students [10]. According to the report of the state examination of autumn 2022, released by the German National Institute for state examinations in Medicine, Pharmacy, and Psychotherapy, 26,7% of international students failed the exam. Comparatively, only 9,5% of the German students failed the exam [11]. Finally, international medical students are characterised by higher drop-out rates during their studies, which may mirror the cumulative burden resulting from the various financial, social, and language difficulties [12]. 

Taking these difficulties into account, medical faculties have started to design support programs for international students, especially in their first years of studies [9], [13], [14]. The goal was to provide a safe space for international students to connect with each other while also improving their academic performance [15]. Some of these programs were based on the method of peer-assisted learning (PAL) [4]. PAL is a well-established teaching method in which a tutor is responsible for a group of tutees. The tutor, also a student, is usually older and/or has more knowledge on the topic [16]. Tutees learn new information from their tutor on how to revise the content taught by university professors and apply their knowledge under the tutor’s support [17]. Tutors are able to use a more familiar language when teaching the most relevant topics, which promotes cognitive congruence between tutors and tutees [18]. Further, as tutors have overcome the same difficulties as the tutees, they are able to develop a more empathetic and supportive relationship [19], increasing social congruence amongst one another. There are several further benefits of PAL described in the literature, such as the creation of a sense of camaraderie between tutees and a welcoming learning environment [20]. 

The method of PAL has gained traction in recent years, with it being implemented in a variety of fields. Areas of application in medical education include problem-based-learning [21], gross anatomy [22], skills-lab training [23], [24], history taking [25], emergency medicine [26], as well as mentoring, counselling, and tutoring [27]. So called “buddy programs’’, offered at various universities worldwide, pair international students in higher semesters with first semester students, thereby fostering a more personal mentoring environment [28]. Welcoming programs that provide international students with orientation at the beginning of their studies have been evaluated at the University of Cologne and at the Charité Hospital in Berlin [14], [29]. Weekly peer-assisted tutorials for preclinical international students, which revise the content taught in university courses, have been proven to be a successful tool in supporting international students in the first year of their studies [4]. 

From a methodological evaluation perspective, previous studies on the topic of PAL in the field of international students focused mostly on the qualitative perceptions of tutees attending courses and mentoring programs [14], [21]. In revision courses, students profited from feedback from tutors, teaching materials, and mock exams, leading to an increased perceived confidence and subjective gains in competence [4], [15]. The personal experience of tutors working in PAL programs for international students has also already been analyzed in previous studies [30]. However, there is a lack of research measuring the objective benefits of PAL on international students’ improvement in knowledge. This research gap is even bigger when different medical subjects are taken into consideration. 

The importance of psychosocial medicine in medical curricula has steadily increased over time. In Germany, during the 1970s, psychosomatic medicine and psychotherapy were introduced as part of the medical approbation, including “medical psychology and medical sociology” as pre-clinical subjects [31]. Since then, it has set the foundation for the clinical subjects “psychiatry, psychosomatic medicine and psychotherapy”, further promoting a more empathetic, patient centered approach [32]. In “medical psychology and medical sociology”, students learn relevant aspects about the doctor-patient-relationship, developmental psychology, social aspects of health and disease, prevention, and more [33]. The relevance of these subjects is emphasized by the structure of the first German state examination, in which “medical psychology and medical sociology” represents one fifth of the questions. In German medical faculties, there are various repetition courses for the state examination in subjects such as “physiology”, “biochemistry”, and “anatomy”. However, few of these focus on “medical psychology and medical sociology”. Only the universities of Jena, Marburg, Münster, Essen and Mainz [34], [35], [36], [37], [38] offer repetition courses for the university exam and first state examination in the subjects “medical psychology and medical sociology”. Yet, none of these courses focus on international students. When taking the social, language, and cultural difficulties into consideration, one could assume that international students would profit from a PAL program on the subjects “medical psychology and medical sociology”. However, to the best of our knowledge, no studies have explored the usage of PAL methods for international students in teaching psychosocial medical subjects. 

Given the limited research, we evaluated the effect of a three-day peer-led revision course on the knowledge level of international preclinical medical students in the subjects “medical psychology and medical sociology”. In comparing pre- and post-test results, we expected that students would show an improvement in their test-results and, consequently, in their knowledge on these subjects. 

## 2. Methods

### 2.1. Design of the revision course 

First and second year preclinical international medical students at the University of Heidelberg were invited to take part in a three-day revision course of the subjects “medical psychology and medical sociology”, offered by the “Heidelberg’s Tutorial for International Medical Students” (HeiTiMed) [15]. HeiTiMed is a project that uses PAL as the predominant teaching method. HeiTiMed tutors are international students in their third year or higher, meaning that they have already successfully passed the first German state examination. Furthermore, HeiTiMed tutors provide weekly tutorials to international preclinical students in the subjects “anatomy”, “physiology”, and “biochemistry”, for which they prepare teaching materials. The aim of the three-day revision course was to prepare first year students for their upcoming university exam and second year students for the first German medical state examination in the subjects “medical psychology and medical sociology”. Tutors were responsible for preparing teaching materials and organizing the revision course. The revision course had to be comprised of the most important topics taught at university lectures on the subjects “medical psychology and medical sociology” and include the most frequently asked questions in the first German medical state examination [33]. Even though “medical psychology and medical sociology” are in fact two different subjects, these subjects have been taught at university lectures together and are also questioned together in the state examination. Table 1 (Tab. 1) presents the topics of both subjects that were taught in the three-day revision course. The interested reader is referred to attachment 1 . There, the topics presented in table 1 (Tab. 1) are explained in detail.

The tutors used an interactive approach to teaching, following the sandwich principle of Kadmon, with short lectures including interactive discussions between tutees and tutors [39]. At the end of each session, tutees answered questions from previous state examinations and the answers were discussed together with the tutors.

### 2.2. Participants

The presented study followed a pre-post design. It involved first- and second-year international medicine students during the years 2023 and 2024. The course was only offered in the summer semester, so students were either in their second or fourth study semester (first or second year). The goal of the revision course was to best prepare first-year international students for their upcoming exam in the subjects “medical psychology and medical sociology” and second-year international students for the first German state examination. German students were also welcome to take part in the course. For the purpose of this study, we defined “international students” as students that have a native language that is not German or acquired their university entry qualification outside of Germany. 30 participants matched our definition of an international student (n=30). 

### 2.3. Test design

Before and after the three-day revision course, participants took one test. This test included various sociodemographic questions and ten “medical psychology and medical sociology” multiple-choice questions taken from previous state examinations. These multiple-choice questions were accessed through the digital medical learning platform AMBOSS, a tool that helps students preparing themselves for the state examinations, and offers specific specialist knowledge to graduated physicians [40]. It includes a library and a question bank following USMLE guidelines [41]. We accessed the data bank with Heidelberg’s Medical Faculty license. 

By calculating how often a question is answered either correctly or incorrectly, the platform creates an indicator for the question’s difficulty [42]. Each question has an attributed difficulty, which is signaled by hammers. These range from: very easy – one hammer (easiest 20% of questions), easy – two hammers (20% of the questions are easier, 50% are more difficult), intermediate – three hammers (50% of the questions are easier, 20% are more difficult), difficult – four hammers (80% of questions are easier, 5% are more difficult), very difficult – five hammers (most difficult 5% of questions). We made use of this system and of the available questions to build two different test versions that have the same number of questions of each difficulty level: one very easy question, two easy questions, four intermediate questions, two difficult questions and one very difficult question. To construct the test, we sampled four times the necessary number of questions for each difficulty and at least four questions of each topic of the subjects “medical psychology and medical sociology”. Then, we chose at least one question of each topic for both versions of the test. These versions were named A and B. All of the questions had only one correct answer.

### 2.4. Procedure 

Students were randomly assigned a test version as their pre-test. All tests were written on paper. After the revision course, participants took the other version as post-test. Group 1 was defined as students that wrote version A as pre- and B as post-test, while group 2 consisted of students who wrote version B as pre- and A as post-test. 

### 2.5. Ethics

This study was conducted following the principles of the Declaration of Helsinki (64^th^ WMA General Assembly, Fortaleza, Brazil, October 2013). Study participation was voluntary. All participants were informed about the purpose of this study and were ensured of the anonymity of their data. Written informed consent was given by all participants. Ethics approval was granted by the ethic committee of the University of Heidelberg (Ethics approval No. S-535/2016). 

### 2.6. Statistical analysis 

Data analysis was conducted using the statistics software R [43]. The handwritten tests were scored by the first author, with each correct answer assigned one point. The points were then summed up to a total number of correct answers, meaning that a student could reach up to 10 correct answers in the pre- and post-test. To compare the results of the pre- and post-test and find a possible difference between group 1 and 2, a two-way ANOVA was calculated. Prerequisites, such as identifying outliers, normality, homogeneity of variances, and covariances, were verified beforehand. The outliers were identified through a box plot. The only outlier in the 6^th^ study year (11^th^ semester) was left out of the analysis. Normality was verified with the Shapiro’s Test, whereas the homogeneity of variance was verified with the Levene’s Test. For the verification of the homogeneity of covariances, a Box’s M-Test was conducted [44]. 

### 2.7. Power analysis

We conducted an a priori power analysis for the ANOVA with G*Power 3 [45]. With an estimated medium effect size f=0.25, level of significance α=0.05, power β=0.8 and an estimated correlation among repeated measures r=0.5, we calculated a required sample size of 34 participants, with 17 participants in each group. After two years of data collection, we achieved a sample size of 30 participants.

## 3. Results

### 3.1. Sample description

Following the exclusion of participants who were native German speakers and had completed their university entrance qualification in Germany, there were n=30 participants left that matched our definition of an “international student”. Of these 30 participants, 2 participants took part in the post-test only and did not provide us with their sociodemographic data. As four participants did not take part in the post-test, their test scores were not considered in the ANOVA calculation. Therefore, 24 international participants (80%) were included in the analysis. 

Of the 28 international participants that provided us with their sociodemographic data, 20 were female and 8 were male. Mean age was 19.6±1.1 years. 15 participants were in the first year (2^nd^ semester) and 13 participants in the second year (4^th^ semester). Most international students were from Eastern Europe (10 students), followed by Turkey (6 students), and East Asia (4 students). Three students came from Russia and three from Latin and North America. The remaining three students were from Western Europe, the Middle East, and Southeast Asia, respectively. 

### 3.2. Comparison of the results between pre- and post-test and between groups 

**Figure 1:**
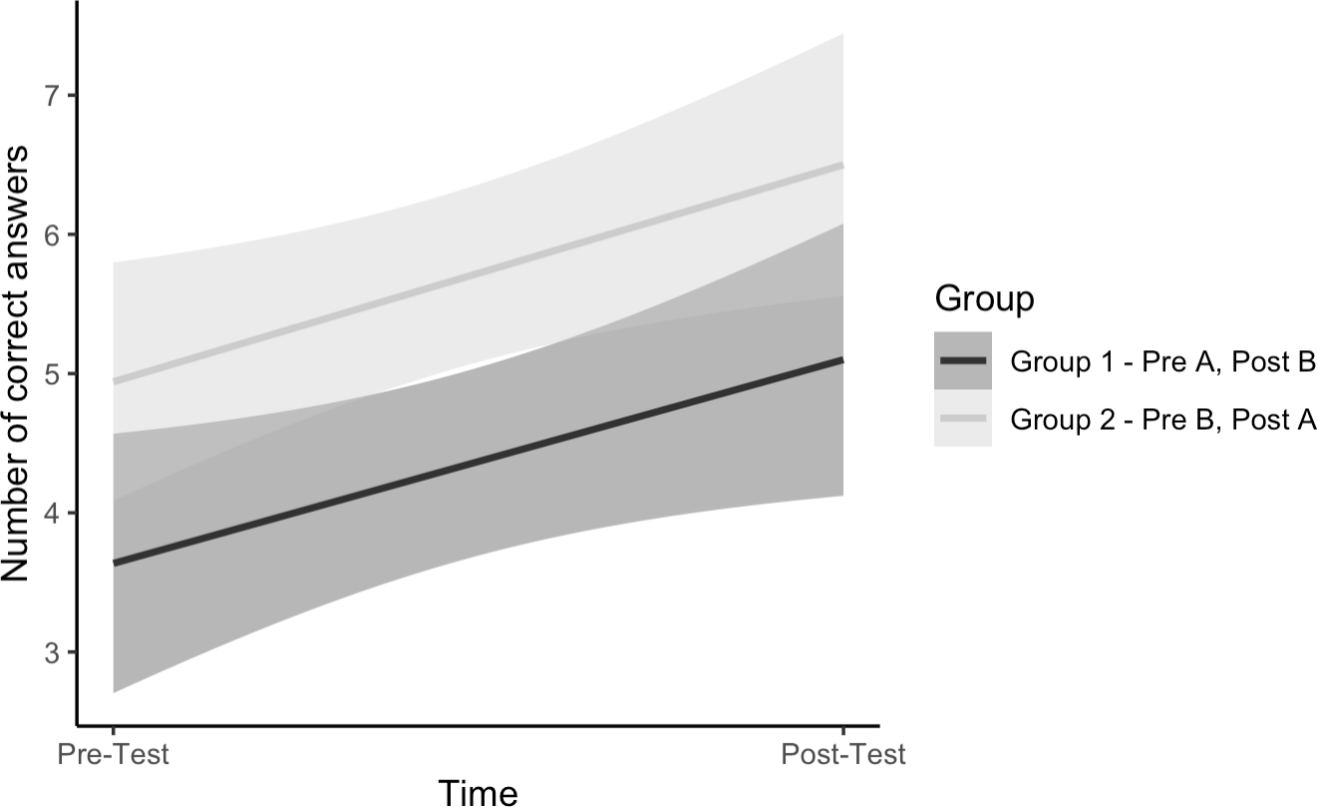
Improvement of correct answered questions between pre- and post-test. The Figure shows the means and standard deviations (grey colored) of the number of correct answers for group 1 and group 2 in pre- and post-assessments.

As shown in table 2 (Tab. 2) and figure 1 (Fig. 1), a significant difference in correctly answered questions between pre- and post-test was found. On average, post-test results were higher by 1.5 correctly answered questions. The mean for the number of correct answers in group 1 was 3.6±1.4 in the pre-test and 5.1±1.5 in the post-test. For group 2, the mean was 4.9±2.0 in the pre-test and 6.5±1.3 in the post-test. There is a significant difference between groups. 

## 4. Discussion

To our knowledge, there are very few studies that have investigated the impacts of PAL on international students’ knowledge. Furthermore, to the best of our knowledge, no other study has investigated psychosocial medicine as the teaching subject for international students. Our results show that international students significantly improved their knowledge after attending a PAL based revision course in “medical psychology and medical sociology”.

These results align with the general benefits of PAL described in the literature [20]: With the concise and comprehensible learning material provided, and together with the tutors’ explanations, tutees received a structured repetition of the previous content. This may have made it easier for international students to remember the previously learned topics [15]. With the tutors’ guidance, international students could have been able to focus more on the most important topics, possibly reducing the time invested in independent study [15]. 

Tutors, being international students themselves, might have a greater understanding regarding language difficulties. This could have led to a tendency of reducing the use of technical terms and therefore making use of a more understandable and approachable language [20], [46]. Even though tutors and students may have different cultural backgrounds, they share similar experiences by being international students. Therefore, tutors could have had a deeper understanding of tutees’ needs and difficulties, as they themselves have overcome similar challenges in early stages of their studies [18]. By additionally possessing knowledge on the topic being taught, tutors may have been better equipped to develop teaching strategies that improve tutees’ understanding, therefore enhancing the cognitive congruence between them [19]. Such strategies could show a more understanding and empathetic approach towards tutees’ specific needs and difficulties [18]. By having a similar social role as tutees, tutors might have been able to craft a more friendly and less formal relationship with tutees, so that a more comfortable learning environment could be established [20], [45]. 

Furthermore, international students were encouraged to actively participate by answering tutors’ direct questions. This interaction between tutor and tutees could have promoted a more active engagement from tutees with the topic, as international students were regularly encouraged to test their own knowledge throughout the revision course. This active participation could have helped students in paying attention to the revision course for longer, which, consequently, could have facilitated knowledge acquisition [47]. At the end of each presentation topic, international students answered multiple-choice questions and discussed answers with tutors. This could have helped international students develop a more efficient and successful strategy for answering multiple-choice questions [17]. 

Motivating students to actively participate in the course by sharing their knowledge through question answering might have allowed tutors to act as facilitators of knowledge acquisition. This may have lessened the hierarchical relationship, encouraging tutors to be seen as facilitators rather than authorities [48]. Furthermore, students may have felt more confident to ask their questions freely during the revision course, leading to a better understanding of the contents. 

Even though there was a difference between group 1 and 2, which can be explained by different individual knowledge levels, both groups objectively improved their knowledge. We were able to show this improvement in the field of psychosocial medicine, with international students learning more about human behaviour, psychotherapy methods, doctor-patient-relationship, and communication. The intense exposure to such topics early in their medical studies, supported by the extracurricular revision course, could be beneficial to international students’ future career by leading them to take a more empathetic and thoughtful approach towards patients. 

Taking into consideration the fact that medical faculties in Germany have reported challenges in supporting their international students [9], we believe that a broader implementation of PAL based tutorial programs for international students could be an effective way of alleviating these difficulties. Programs, such as the revision course in this study, HeiTiMed with weekly tutorials and social gatherings [4], the “buddy programme”, and language orientation courses [14] have proven to be effective in supporting international students academically and socially. It would also be interesting to implement PAL as a teaching tool for practical skills, such as drawing blood and performing physical exams. In the field of psychosocial medicine, this method could be even more valuable as a tool to support the development of a more empathetic and patient-centred communication. International students could further develop their practical and communication skills by participating in additional practice sessions with a simulated patient under the guidance of a tutor.

### 4.1. Limitations 

The major limitation of the current study is a missing control group of students who didn’t take part in the revision course. A control group could further support the results, emphasizing the effect of the revision course on international students’ knowledge. The sample size of 30 participants is smaller than the required sample size of 34 participants to achieve a power β=0.8. Furthermore, the observed improvement in international students’ knowledge may not be solely attributable to the PAL method. Other factors, such as the additional time spent engaging with the subjects taught during the revision course, may also have contributed to the post-test results. The voluntary nature of participation in the course could have also led to the selection of a group of highly motivated students, which may have further contributed to the improvement seen in the post-test. Another limitation of our study is that this revision course was taught exclusively by international tutors. As we mostly attribute the success of our results to the social and cognitive congruence between international students and tutors, it would be interesting to replicate our findings with a control group taught by German tutors. Furthermore, the study focused solely on the students’ improvement within the three-day revision course. Long-term outcomes, such as the exam results of the first German medical state examination, are missing.

## 5. Conclusion

To our knowledge, there is a lack of research regarding the effects of PAL in settings with international students. This study assessed the objective improvement in international students' knowledge after attending a three-day peer-led revision course. During this time, international students significantly improved their knowledge in a psychosocial medical subject. These results align with the benefits of PAL described in the literature. The increased cognitive and social congruence between international students and tutors could have created a safe learning environment that may have contributed to international students’ acquisition of knowledge. However, further research on the effects of PAL in settings with international students is necessary. As in this study, we recommend an objective approach that quantifies academic development, accompanied by an analysis of the subjective benefits for international students, following the approach of Huhn et. al. [15]. Moreover, further research is needed to evaluate the long-term effects of PAL on international students’ academic career.

## Data

The datasets generated and analyzed during the current study are available at HeiData under [https://doi.org/10.11588/DATA/1STVOK] [49].

## Abbreviations

- HeiTiMed: Heidelberg’s Tutorial for International Medical Students
- PAL: Peer-assisted learning

## Notes

### Ethics approval

Ethics approval was granted by the Ethics Committee of the University of Heidelberg: No. S-535/2016. 

### Consent to participate

Study participation was voluntary. All participants were informed about the purposes of this study and were ensured anonymity of their data. All participants provided their written consent. 

### Funding

Heidelberg’s Tutorial for International Medical Students (HeiTiMed) is a project funded by Heidelberg’s Medical Faculty. 

### Author contributions 

CN and ID conceived and designed the study. ID supervised the revision course. JS was a tutor teaching the course. JS and ID carried out the statistical analysis. JS drafted the manuscript, which was revised by ID, CN and HCF. All authors read and approved the final manuscript. 

### Authors’ ORCIDs

- Julia Sgrott: [0009-0002-4888-985X]
- Christoph Nikendei: [0000-0003-2839-178X]
- Hans-Christoph Friederich: [0000-0003-4344-8959]
- Ivo Dönnhoff: [0000-0003-1538-9971]

## Acknowledgements

We thank the tutors Andreas Royer, Hagyu Thomas Seong, Elizabeth Tong, Aleksei Smirnov, Isabel Hamm-Teixeira dos Santos and Josefina Arias Alvarado of the Heidelberg’s Tutorial for International Medical Students (HeiTiMed), who were responsible for teaching the revision course. Further, we thank Molly Beatrix Sutcliffe for revising this manuscript. 

## Competing interests

The authors declare that they have no competing interests. 

## Supplementary Material

Topic overview revision course

## Figures and Tables

**Table 1:**
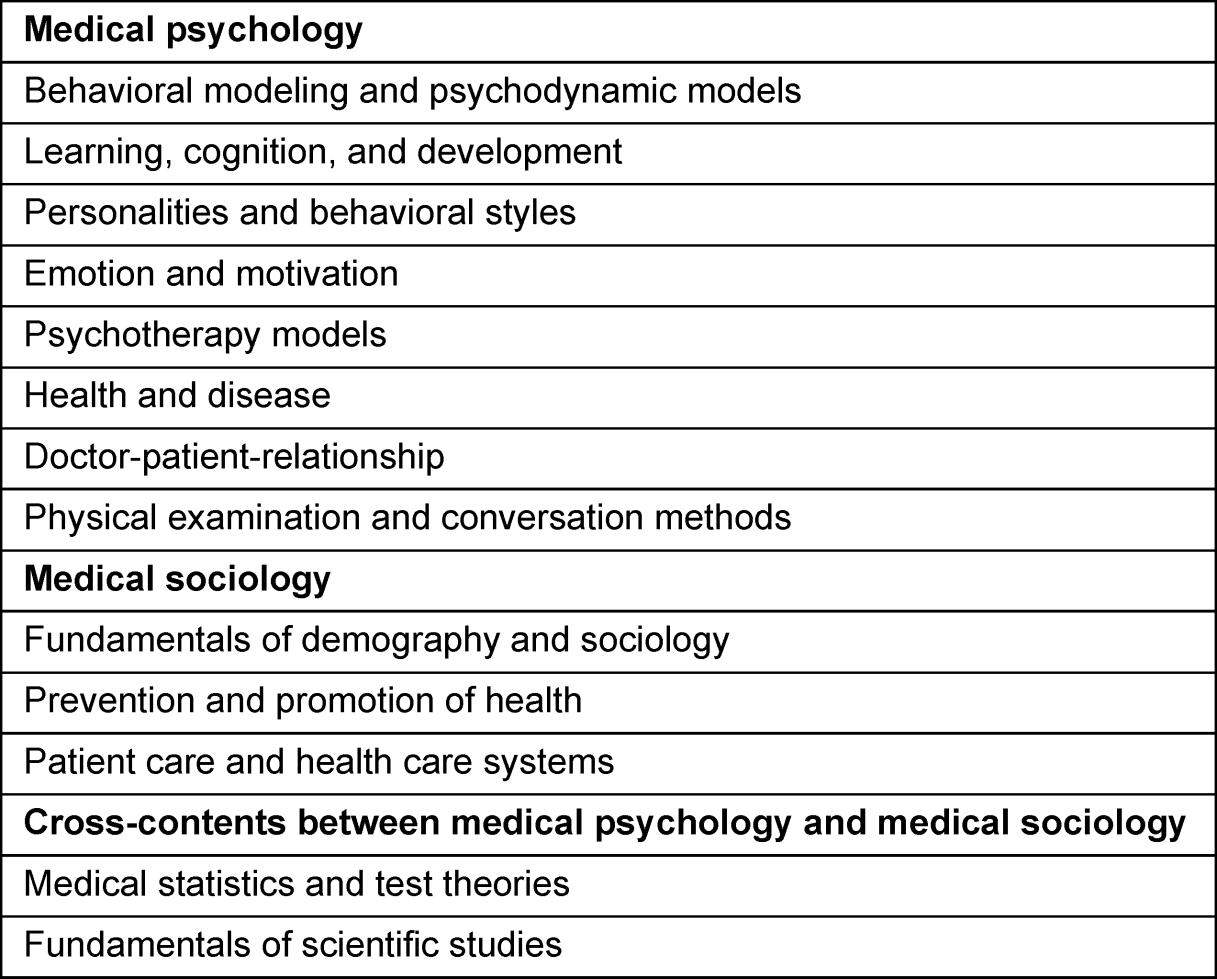
Table of contents of the three-day-revision course

**Table 2:**
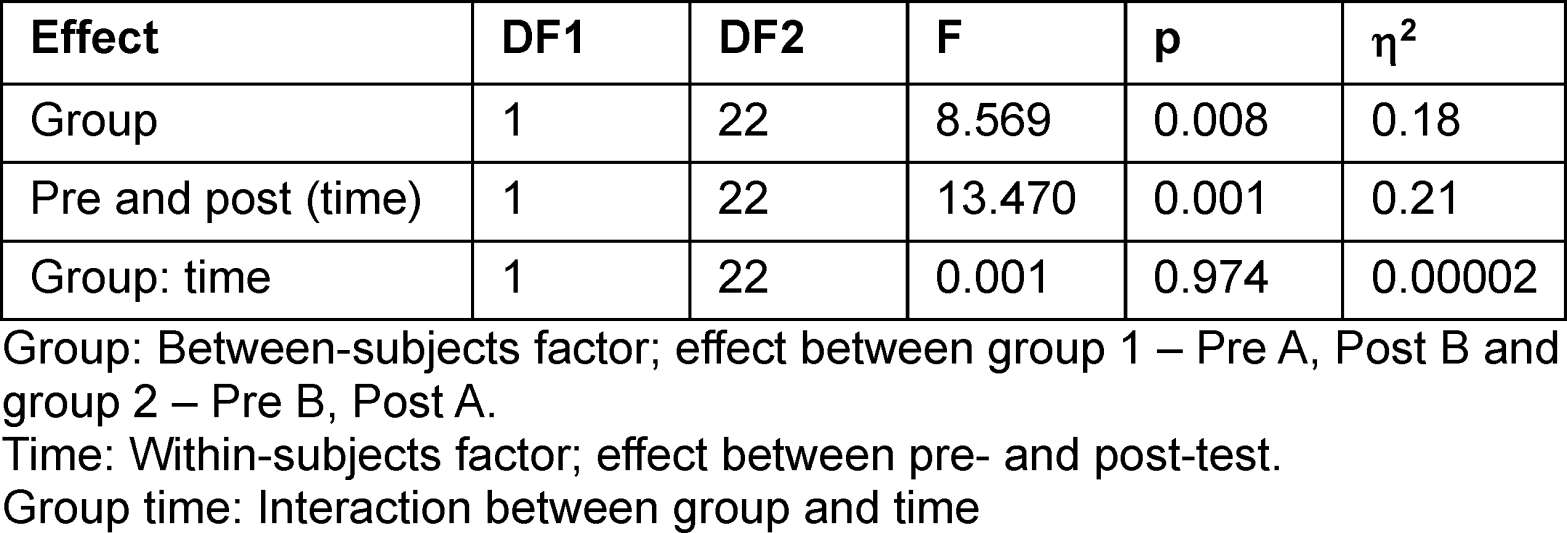
ANOVA for the differences between pre- and post-test results and between groups

## References

[1] Sachverständigenrat deutscher Stiftungen für Integration und Migration (SVR) (2011). Vom internationalen Studierenden zum hoch qualifizierten Zuwanderer. Ein Vergleich der rechtlichen Rahmenbedingungen in fünf Staaten der Europäischen Union.

[2] Statistisches Bundesamt (2024). Statistischer Bericht – Statistik der Studierenden – Wintersemester 2023/2024.

[3] McManus IC, Richards P, Winder BC, Sproston KA, Styles V (1995). Medical school applicants from ethnic minority groups: identifying if and when they are disadvantaged. BMJ.

[4] Huhn D, Al Halabi K, Alhalabi O, Armstrong C, Castell Morley A, Herzog W, Nikendei C (2018). Interactive peer-guided examination preparation course for second-year international full-time medical students: quantitative and qualitative evaluation. GMS J Med Educ.

[5] Sherry M, Thomas P, Chui WH (2010). International students: a vulnerable student population. High Educ.

[6] Huhn D, Schmid C, Erschens R, Junne F, Herrmann-Werner A, Möltner A, Herzog W, Nikendei C (2018). A comparison of Stress Perception in International and Local First Semester Medical Students Using Psychometric, Psychophysiological, and Humoral Methods. Int J Environ Res Public Health.

[7] Sawir E (2005). Language difficulties of international students in Australia: the effects of prior learning experience. Int Educ J.

[8] Andrade MS (2006). International students in English-speaking universities: Adjustment factors. J Res Int Educ.

[9] Huhn D, Junne F, Zipfel S, Duelli R, Resch F, Herzog W, Nikendei C (2015). International medical students - a survey of perceived challenges and established support services at medical faculties. GMS J Med Educ.

[10] Huhn D, Lauter J, Roesch-Ely D, Koch E, Möltner A, Herzog W, Resch F, Herpertz SC, Nikendei C (2017). Performance of International Medical Students in Psychosocial Medicine. BMC Med Educ.

[11] Institut für medizinische und pharmazeutische Prüfungsfragen (IMPP) (2023). Ergebnisse des Ersten Abschnitts der Ärztlichen Prüfung - Herbst 2022.

[12] Huhn D, Resch F, Duelli R, Möltner A, Huber J, Karimian-Jazi K, Amr A, Eckart W, Herzog W, Nikendei C (2014). Examination performances of German and international medical students in the preclinical studying-term--a descriptive study. GMS J Med Educ.

[13] Huhn D, Nikendei C (2018). International students - support and integration initiatives at Medical Faculties in Germany. GMS J Med Educ.

[14] Marmon W, Arnold U, Maaz A, Schumann M, Peters H (2018). Welcome, Orientation, Language Training: a project at the Charité for new international medical students. GMS J Med Educ.

[15] Huhn D, Eckart W, Karimian-Jazi K, Amr A, Herzog W, Nikendei C (2015). Voluntary peer-led exam preparation course for international first year students: Tutees’ perceptions. BMC Med Educ.

[16] Ten Cate O, Durning S (2007). Dimensions and psychology of peer teaching in medical education. Med Teach.

[17] Alexander SM, Dallaghan GLB, Birch M, Smith KL, Howard N, Shenvi CL (2022). What Makes a Near-Peer Learning and Tutoring Program Effective in Undergraduate Medical Education: a Qualitative Analysis. Med Sci Educ.

[18] Lockspeiser TM, O'Sullivan P, Teherani A, Muller J (2008). Understanding the experience of being taught by peers: the value of social and cognitive congruence. Adv Health Sci Educ Theory Pract.

[19] Loda T, Erschens R, Loenneker H, Keifenheim KE, Nikendei C, Junne F, Zipfel S, Herrmann-Werner A (2019). Cognitive and social congruence in peer-assisted learning - A scoping review. PLoS One.

[20] Shenoy A, Petersen KH (2020). Peer Tutoring in Preclinical Medical Education: A Review of the Literature. Med Sci Educ.

[21] Herrmann-Werner A, Gramer R, Erschens R, Nikendei C, Wosnik A, Griewatz J, Zipfel S, Junne F (2017). Peer-assisted learning (PAL) in undergraduate medical education: An overview. Z Evid Fortbild Qual Gesundhwes.

[22] Han ER, Chung EK, Nam KI (2015). Peer-assisted learning in a gross anatomy dissection course. PloS One.

[23] Blohm M, Lauter J, Branchereau JS, Krautter M, Köhl-Hackert N, Jünger J, Herzog W, Nikendei C (2015). "Peer-Assisted Learning" (PAL) in the Skills-Lab – an inventory at the medical faculties of the Federal Republic of Germany. GMS J Med Educ.

[24] Weyrich P, Celebi N, Schrauth M, Möltner A, Lammerding-Köppel M, Nikendei C (2009). Peer-assisted versus faculty staff-led skills laboratory training: a randomised controlled trial. Med Educ.

[25] Nikendei C, Andreesen S, Hoffmann K, Junger J (2009). Cross-year peer tutoring on internal medicine wards: effects on self- assessed clinical competenciese - A group control design study. Med Teach.

[26] Jauregui J, Bright S, Strote J, Shandro J (2017). A Novel Approach to Medical Student Peer-assisted Learning Through Case-based Simulations. West J Emerg Med.

[27] Olaussen A, Reddy P, Irvine S, Williams B (2016). Peer-assisted learning: time for nomenclature clarification. Med Educ Online.

[28] Nillson P (2019). The Buddy Programme: Integration and Social Support for International Students. J Comp Int High Educ.

[29] Karay Y, Restel K, Marek R, Schlüter de Castro B (2018). Studienstart International of the University of Cologne: The closely supervised semester of study entry for students from third countries using the example of the model degree program for human medicine. GMS J Med Educ.

[30] Delly P (2021). "I feel a deep sense of belonging to the team": International student experiences as peer-assisted learning advisers. J Acad Lang Learn.

[31] Köllner V (1995). Die Ausbildung im Fach Psychosomatische Medizin und Psychotherapie in der Bundesrepublik Deutschland. Ther Umschau.

[32] Schüppel R, Bayer A, Hrabal V, Hölzer M, Allert G, Tiedemann G, Hochkirchen B, Stephanos S, Kächele H, Zenz H (1998). Fachübergreifendes Längsschnittcurriculum "Medizinische Psychologie, Psychotherapie und Psychosomatik." Erfahrungen aus dem vorklinischen Abschnitt Psychotherapie, Psychosomatik, medizinische Psychologie. Psychother Psychosom Med Psychol.

[33] Institut für medizinische und pharmazeutische Prüfungsfragen (IMPP) (2005). IMPP Gegenstandskatalog (IMPP-GK1) für den schriftlichen Teil des Ersten Abschnitt der Ärztlichen Prüfung (ÄAppO vom 27. Juni 2002) - Teil ,,Medizinische Psychologie und Medizinische Soziologie’’.

[34] Universitätsklinikum Jena, Institut für Psychosoziale Medizin PuP Medizinische Psychologie und Medizinische Soziologie - Repetitorium.

[35] Universität Münster, Institut für Medizinische Psychologie und Systemneurowissenschaften Studium.

[36] Universitätsmedizin Essen, Institut für Medizinische Psychologie und Verhaltensimmunbiologie Lehre.

[37] Philipp Universität Marburg, Institut für Medizinische Psychologie Übersicht der Lehrangebote der Medizinischen Psychologie.

[38] Johannes Gutenberg Universität Mainz (2016). 04.107. Tutorium Med. Psychologie und Med. Soziologie.

[39] Kadmon M, Strittmatter-Haubold V, Greifeneder R, Ehlail F, Lammerding-Köppel M (2008). Das Sandwich-Prinzip - Einführung in Lerner zentrierte Lehr-Lernmethoden in der Medizin. Z Evid Fortbild Qual Gesundhwes.

[40] Amboss GmbH About us.

[41] Amboss GmbH About AMBOSS.

[42] Amboss GmbH Question difficulty.

[43] R Core Team (2024). R: A Language and Environment for Statistical Computing 2024.

[44] Data Novia Comparing multiple means in R.

[45] Faul F, Erdfelder E, Lang AG, Buchner A (2007). G*Power 3: a flexible statistical power analysis program for the social, behavioral, and biomedical sciences. Behav Res Methods.

[46] Guraya SY, Abdalla ME (2020). Determining the effectiveness of peer-assisted learning in medical education: A systematic review and meta-analysis. J Taibah Univ Med Sci.

[47] Alhawiti NM (2023). The Influence of Active Learning on the Development of Learner Capabilities in the College of Applied Medical Sciences: Mixed-Methods Study. Adv Med Educ Pract.

[48] Yusofi M, Zarghami-Hamrah S, Ghaedy Y, Mahmudnia A (2018). A quasi-transcendental approach for removing hierarchical teacher-student relation. Policy Futur Educ.

[49] Sgrott J, Nikendei C, Friederich HC, Dönnhoff I (2025). Peer-led revision course of the subject ‚Medical Psychology and Sociology‘ for preclinical international medical students [data].

