# Supplementary material for: Enhancing the performance of preclinical international medical students’ in the subjects “medical psychology and medical sociology” through a peer-led revision course
Source: GMS J Med Educ. 2026 Jun 15;43(5):Doc59. doi: 10.3205/zma001853 (PMC13316371; doi:10.3205/zma001853)
Supplement: Topic overview revision course [file JME-43-59-s-001.pdf]

Attachment 1: Topic overview revision course

In this attachment, the topics presented in table 1 are explained in more detail. In addition, the time invested into each topic during the revision course is shown in brackets.

Medical psychology

Behavioral modeling and psychodynamic models (90 min): Learning theory modeling, cognitive modeling and cognitive-behavioral modeling were the behavioral modeling theories presented at the repetition course. The presented psychodynamic models are based on Sigmund Freud’s topographical model and structural model.

Learning, cognition, and development (30 min): Learning theories, such as Pavlovian conditioning and operant conditioning, were taught during the course. Under the topic ‘cognition’, concepts such as perception, memory and intelligence were defined. Furthermore, development theories, such as Piaget’s theory of cognitive development and Kohlberg’s theory of moral development, were revised.

Personalities and behavioral styles (30 min): Topic of the repetition course were Eysenck’s personality theory and the Five-Factor Model.

Emotion and motivation (30 min): The four components of emotion were defined as physiological appraisal, cognitive appraisal, subjective experience, and expressive behaviors. Furthermore, primary and secondary emotions were differentiated. Theories of emotion, such as James-Lange theory, Cannon-Bard theory and Schachter-Singer theory were further explained. The concept of motivation, its hierarchy by Maslow and its conflicts were also defined.

Psychotherapy models (45 min): Psychotherapy models were separated in psychodynamic psychotherapy and behavioral psychotherapy. To psychodynamic psychotherapy models belong Freud’s classic psychoanalysis and depth psychology-based psychotherapy. Behavioral psychotherapy consisted of exposition training, behavioral analysis, and the cognitive-behavioral therapy.

Health and disease (45 min): The WHO-definition for ‘health’ was presented. Further concepts such as quality of life, patient satisfaction, psychosocial risk factors, and protective factors for disease were explained.

Doctor-patient-relationship (90 min): Communication aspects, such as verbal and non-verbal communication and asymmetry between speakers, were introduced. Carl Roger's client-centered therapy model was also explained. More attention was brought to sociocultural interaction difficulties. The different models of the doctor-patient-relationship, such as the paternalistic model, the informative model and shared-decision making, were presented. The relevance of the subjective illness theory and causal attribution was highlighted.

Physical examination and conversation methods (60 min): International students revised the structure of an anamnesis and important aspects of the physical examination, such as the different perspectives between doctors and patients in this type of situation.

Medical sociology

Fundamentals of demography and sociology (30 min): Important demography concepts, such as mortality, lethality, fertility rate, and old-age dependency ratio, were defined. The demographic transition model was further explained. Other relevant topics, such as migration and social inequality, were approached.

Prevention and promotion of health (30 min): All three types of prevention (primary, secondary, tertiary) and prevention measures were presented. Models that promote health, such as the health-belief-model, theory of planned behavior, social-cognitive theory and transtheoretical model, were explained. The cognitive dissonance theory was also approached.

Patient care and health care systems (30 min): The different types of health care systems, such as the Bismarck model and the Beveridge model, were explained. The health care system in Germany was explained in detail.

Cross-contents between medical psychology and medical sociology

Medical statistics and test theories (60 min): Concepts, such as variables, scales, positional measures, means and standard deviation, were defined. Psychometric criteria, such as standardization, reliability and validity, were also explained. Other concepts, including sensitivity, specificity, positive predictive value and negative predictive value, were defined. Epidemiology risks, such as absolute and relative risks, odds ratio, absolute, and relative risk reduction and number needed to treat, were explained.

Important concepts for statistical tests, including p-value, significance level, power, effect size and correlation, were approached.

Fundamentals of scientific studies (30 min): The basic structure of developing a scientific study, composed by hypothesis formation, definition of study type, definition of sampling method and evaluation of the collected data, was presented to international students.

Attachment 1 to Sgrott J, Nikendei C, Friedrich HC, Dönnhoff I. *Enhancing the performance of preclinical international medical students' in the subjects "medical psychology and medical sociology" through a peer-led revision course*. GMS J Med Educ. 2026;43(5):Doc59. DOI: 10.3205/zma001853
